# The effects of radiation therapy on the macrophage response in cancer

**DOI:** 10.3389/fonc.2022.1020606

**Published:** 2022-09-29

**Authors:** Callum Beach, David MacLean, Dominika Majorova, James N. Arnold, Monica M. Olcina

**Affiliations:** ^1^ Department of Oncology, Medical Research Council Oxford Institute for Radiation Oncology, University of Oxford, Oxford, United Kingdom; ^2^ School of Cancer and Pharmaceutical Sciences, King’s College London, London, United Kingdom

**Keywords:** radiotherapy, tumor microenvironment, hypoxia, extracellular matrix, macrophage polarization, macrophage recruitment, tumor associated macrophages (TAM), complement system

## Abstract

The efficacy of radiotherapy, a mainstay of cancer treatment, is strongly influenced by both cellular and non-cellular features of the tumor microenvironment (TME). Tumor-associated macrophages (TAMs) are a heterogeneous population within the TME and their prevalence significantly correlates with patient prognosis in a range of cancers. Macrophages display intrinsic radio-resistance and radiotherapy can influence TAM recruitment and phenotype. However, whether radiotherapy alone can effectively “reprogram” TAMs to display anti-tumor phenotypes appears conflicting. Here, we discuss the effect of radiation on macrophage recruitment and plasticity in cancer, while emphasizing the role of specific TME components which may compromise the tumor response to radiation and influence macrophage function. In particular, this review will focus on soluble factors (cytokines, chemokines and components of the complement system) as well as physical changes to the TME. Since the macrophage response has the potential to influence radiotherapy outcomes this population may represent a drug target for improving treatment. An enhanced understanding of components of the TME impacting radiation-induced TAM recruitment and function may help consider the scope for future therapeutic avenues to target this plastic and pervasive population.

## Introduction

**Figure 1:**
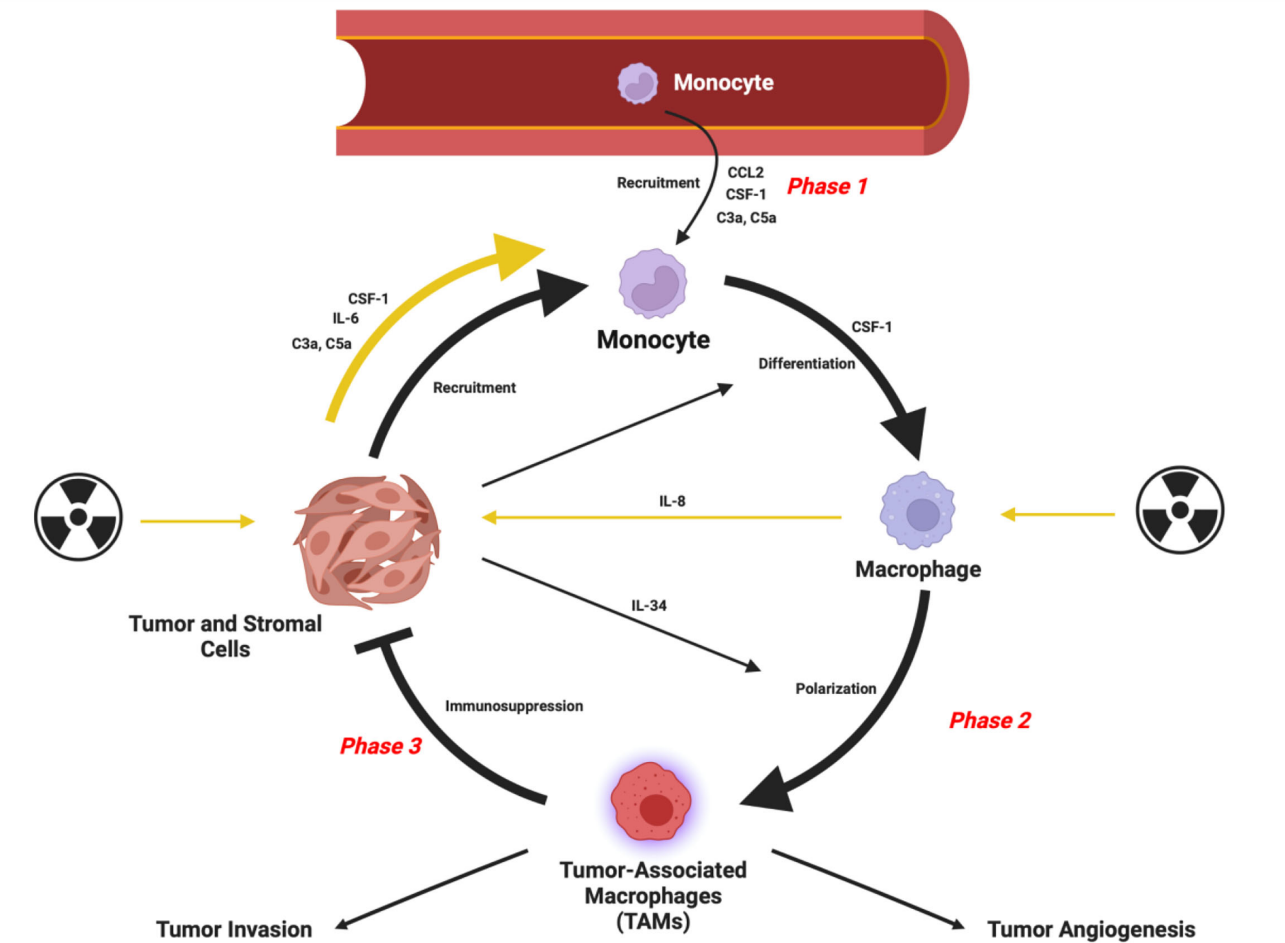
Schematic representation of the role of TAMs in tumor progression. Radiation can contribute to recruitment and polarization as indicated by the yellow arrows. Figure created in Biorender. Agreement number: RO24DGQPW4.

Within neoplastic lesions, immune and mesenchymal cells interact with malignant tumor cells and influence many facets of tumor progression (1–3). Tumor-associated macrophages (TAMs) often make up a large proportion of the immune cell population within the TME. Macrophages are a highly plastic immune cell population, and their phenotypes are shaped by the microenvironments in which they reside (4, 5). In the context of cancer, macrophages are exploited by the tumor cells to adopt phenotypes which counterintuitively, help facilitate disease progression through providing a suitable microenvironment for the progression of multiple carcinomas (6). It is possible to consider the role of TAMs in tumor progression as occurring in phases (**Figure 1**) which include initial recruitment of TAM progenitors, subsequent polarization to an immunosuppressive phenotype and prevention of anti-tumor immune responses. TAMs can also facilitate angiogenesis to meet the metabolic demands of the cancer while assisting the passage of tumor cells into circulation and setting up the site for secondary tumor growth (7–9). Interestingly, the TAM population is phenotypically diverse to the extent that both pro- and anti-tumoral phenotypes of these cells can reside in the same tumor (10, 11). The prevalence of the TAM population correlates with poor patient prognosis in all cancers (except colorectal) (12–14) highlighting this population as a potential therapeutic target in cancer.

Radiotherapy is still a mainstay of cancer treatment for approximately 50% of all cancer patients. It is increasingly recognized that radiotherapy is a strong immune modulator, with the capacity to induce both pro- and anti-inflammatory processes (15, 16). As such, radiation can elicit macrophage recruitment into the tumor (17–20). TAM polarization away from tissue-protection and towards anti-tumoral/immunostimulatory functions could be a potential approach to boost the anti-cancer effects of radiotherapy and capitalize on the immune-stimulating effects of this treatment (16, 19). Here, the effect of radiation on TAM recruitment and polarization will be described. We will particularly focus on changes to soluble and physical components of the tumor microenvironment (TME) which may limit the positive effects of radiation on macrophage plasticity and highlight key examples that could be therapeutically targeted to improve radiation response.

## Phase 1: Recruitment of TAMs

### Recruitment overview

TAMs within the tumor are either present as tissue-resident macrophages or are formed after circulating monocytes are recruited and subsequently polarized into mature TAMs (21, 22). Resident macrophages are present during embryonic development and tend to exist in specific tissues such as Kupffer cells in the liver, and alveolar macrophages in the pulmonary alveolus of the lungs (23). These macrophages can provide a pro-tumorigenic niche and assist with initial tumor growth from a very early stage (24).

### Soluble factors impacting TAM recruitment following radiotherapy

Soluble factors that mediate mobilization are critically associated with recruiting monocytes/macrophages to the TME (**Figure 1**). A well-documented signaling molecule involved in this process is chemokine (C-C motif) ligand 2 (CCL2, also known as monocyte chemoattractant protein 1; MCP1) (25–27). Radiotherapy is known to induce the expression of CCL2 within the TME (28, 29). Increased CCL2 expression can also be regulated by components of the humoral arm of innate immunity such as the complement system (30–32) and the long pentraxin PTX3 (33). Both of these innate immunity components appear to work in concert since PTX3 deficiency results in complement-dependent TAM recruitment in 3-Methylcholanthrene carcinogenesis models (33). Signaling of complement anaphylatoxins C3a and C5a through their respective receptors, C3aR and C5aR1, has been further demonstrated to result in TAM recruitment and polarization towards an immunosuppressive phenotype (30, 31). This includes reduced CD206 expression and upregulation of CD11c, major histocompatibility complex class II, CD80 and CD86 in TAMs from C3 and C3aR1^-/-^ mice (32). Interestingly, expression of C3a, C5a and their receptors C3aR and C5aR1 is induced in melanoma murine tumors following irradiation (20 Gy) (34). Furthermore, complement inhibition at the level of C3 (with a CR2-Crry fusion protein) in combination with radiation has been demonstrated to enhance the numbers of macrophages with an M1-like phenotype (F4/80^+^, CD11c^+^, CD206^-^) in lymphoma tumor models (35).

In addition to chemokines and complement soluble factors, cytokines are also involved in the recruitment of monocytes/macrophages to the TME. Colony-stimulating factor 1 (CSF-1, also known as macrophage colony stimulating factor; M-CSF), which typically is associated with a differentiation/survival signal for monocyte/macrophages, also has chemotactic properties for the recruitment of these cells to a site of inflammation (36, 37). In several tumor types and murine models, radiation has been demonstrated to induce CSF-1 production which can facilitate macrophage recruitment (17, 18). Following irradiation of tumors the DNA damage-induced kinase ABL1 (c-Abl) is recruited into the nuclei of tumor cells to enhance CSF1 transcription (38). CSF-1 production is also induced in response to IL-8, which can be secreted by the macrophages themselves, contributing to a positive feedback axis further perpetuating macrophage recruitment. However, this axis is not necessarily macrophage-specific as cancer cells can also produce IL-8 themselves post-irradiation (39). IL-34 is a cytokine that shares its receptor with CSF-1, binding CSF1-R, and as such they have similar biological properties. Like CSF-1, IL-34 expression is induced after irradiation (40). This induction has also been demonstrated to promote monocyte recruitment to the TME and subsequent polarization to an immunosuppressive phenotype (41).

Furthermore, tumor cells produce IL-6 in response to radiation-induced damage which promotes monocytes/macrophage recruitment to the TME (42–44). In a double-edged role for IL-6, once monocyte recruitment occurs, the cytokine also blocks dendritic cell differentiation and promotes monocytes to differentiate towards a TAM-like cell with an immunosuppressive phenotype (6, 45).

### Physical changes in the TME affecting TAM recruitment following radiotherapy

Hypoxia (low oxygen tension) is a common physical feature of the TME that arises due to insufficient oxygen supply to support rapidly growing tumors. Hypoxia is particularly relevant to radiotherapy since cells irradiated under reduced oxygen levels are more resistant to the lethal effects of radiation (46). Hypoxia-inducible factors (HIFs) are key to the transcriptional response to hypoxia. HIF heterodimers consist of an oxygen-sensitive subunit (HIF-1α, HIF-2α or HIF-3α), and a constitutively expressed HIF-β subunit. Under ambient oxygen concentrations, HIF-α subunits are continually degraded by ubiquitination and proteasomal degradation. However, under low oxygen tensions, HIF-α subunits are stabilized and trafficked to the nucleus where they modulate gene expression through binding hypoxia-responsive elements of specific genes associated with the hypoxic response (47–49). Both HIF-1α and HIF-2α can accumulate in macrophages exposed to hypoxic conditions *in vitro* (50, 51). *In vivo*, HIF-1α has been found to be essential for maintenance of appropriate cellular ATP pools necessary for myeloid cell motility and function (52). Furthermore, following tumor irradiation, nitric oxide (NO) generation in TAMs results in s-nitrosylation of HIF-1α at its oxygen-dependent degradation domain which prevents its destruction. Pharmacological inhibition of NO production is associated with reduced tumor growth following irradiation (53). Furthermore, studies using mice specifically lacking HIF-2α in myeloid cells have demonstrated reduced TAM infiltration in hepatocellular and colitis-associated colon carcinoma models through regulation of cytokine receptor CSF-1R and chemokine receptor CXCR4. Interestingly, this observed reduction in TAM infiltration was associated with reduced tumor cell proliferation (54). HIF-dependent induction of CCL2 also further supports monocyte/macrophage recruitment (55). A recent study has demonstrated that vascular endothelial growth factor-A (VEGF-A), another HIF-regulated gene, also plays a key role in both the recruitment of macrophages and the polarization toward an immunosuppressive phenotype as shown by the increase of the marker CD163 (56).

### Extracellular matrix

The extracellular matrix (ECM), which constitutes the protein scaffold around the tumor and stromal cells, has a role in providing a platform for innate immune cell infiltration, with many of its components and post-degradation fragments sharing the ability to recruit monocytes. Much focus has been directed to proteolytic fragments of the ECM which have been demonstrated to represent endogenous ligands for binding and activating toll-like receptors (TLRs). The release of glycosaminoglycan hyaluronan (HA) after irradiation of the tumor has been documented (57). HA can also play a role in facilitating macrophage infiltration into the tumor stroma through an interaction with the HA receptor CD44 expressed by macrophages (58). Monocytes/TAMs recruited by the CD44:HA axis have an immunosuppressive phenotype. This is facilitated by the upregulation of IL-10 expression while concurrently downregulating NF-κB signaling (59).

In addition to HA, latent TGF-β (an inactive form of the cytokine) is also released by the ECM post-irradiation. Once activated, TGF-β has a potent influence on TAM recruitment. This can occur directly through enhanced integrin expression and type IV collagenase secretion (60) and indirectly through the upregulation of CXCR4 on monocytes, with perivascular fibroblast expression of CXCL12 attracting the monocytes to the tumor bed (61).

Additionally, damaging the ECM leads to macrophage recruitment due to the attraction of immunosuppressive TAMs through the scavenger receptor CD206 (mannose receptor). This allows the phagocytosis and degradation of collagen fragments to form a strong chemoattractant for macrophages (62, 63). This leads to a feedback loop where initial radiation-induced damage to the ECM leads to recruitment of TAMs that themselves facilitate a continuous wound-healing state within the tumor site, further increasing monocyte/TAM recruitment. In a similar fashion, elastin fragments generated by the activity of macrophage-derived MMPs (9 and -12) have been demonstrated to act as chemotactic factors for monocytes, creating a positive feedback loop which increases the prevalence of TAMs in the TME (64).

## Phase 2: Macrophage polarization

### Polarization overview

**Figure 2:**
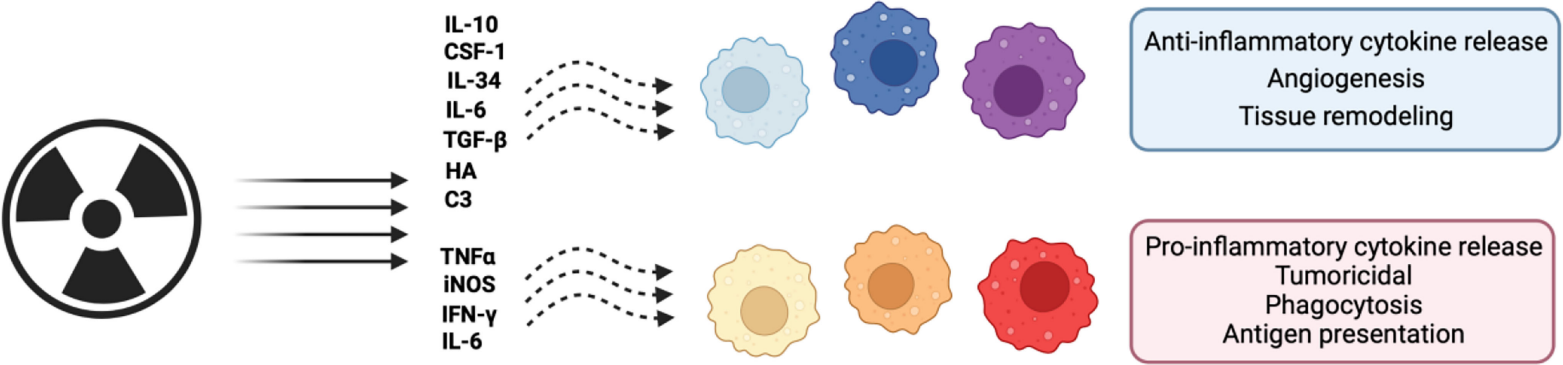
Schematic representation of the effects of radiation on macrophage polarization. Macrophages can adopt both pro- and anti-tumoral phenotypes across a spectrum of possible polarization states. Shown are the effects of radiation on these phenotypes and effector molecules. Figure created in Biorender. Agreement number: LH24DGQ49Q.

Previously, monocyte polarization into mature macrophages was thought to be binary, with TAMs either acting as inflammatory or immunosuppressive agents within the stroma (65). However, it is becoming increasingly clear that, once polarized, the TAMs phenotypically fall on a spectrum (4). Data, mostly gathered from *in vitro* studies, has indicated that polarization on this spectrum may depend on the presence of specific factors such as IL-4, IL-10, IL-13, IFNγ, and lipopolysaccharide (LPS) (66, 67) (**Figure 2**). Once these factors bind to their respective receptor, monocytes undergo polarization and maturation into more specialised TAM phenotypes through downstream signal transduction pathways altering transcription within these cells (68). Recently, it has been identified that TAM polarization can be refined to a three-way polarization program in a spontaneous murine model of breast cancer (11). This three-way program is broadly split into an alternatively-activated-like, angiogenic/immunosuppressive, and inflammatory phenotypic specialization of these cells (11).

### Pathways involved in radiation-induced polarization

Following irradiation, macrophage polarization towards either pro- or anti-inflammatory sides of the spectrum may be dependent on irradiation dose and which transcription factors are formed to drive downstream gene expression (69, 70). NF-κB is a key modulator of macrophage polarization and NF-κB p65-p50 heterodimers can initiate transcription of pro-inflammatory genes such as TNFα, IL1β, IL6, IL12, IFNγ and CXCL10 (70). Increased p65/RelA expression following 2 Gy irradiation of the RAW264.7 macrophage cell line or CD11b^+^ peritoneal macrophages, is associated with increased levels of inducible nitric oxide synthase (iNOS, which is an M1-associated marker) (71). Low dose (2 Gy) whole body irradiation has also been demonstrated to induce iNOS, and concurrently reduce M2-associated markers such as Ym-1 and Fizz-1 in peritoneal macrophages. iNOS expressing TAMs in turn appear important for effector T-cell recruitment into the tumor through vascular normalization (69). Irradiation of human monocyte-derived macrophages with 2, 6 or 10 Gy, results in increased RelB expression which is accompanied by reduced expression of anti-inflammatory genes (such as CD163, and IL-10) (72). Conversely, loss of NF-κB p50 expression has been associated with a pro-inflammatory macrophage phenotype including enhanced TNFα and reduced IL10 expression in bone marrow-derived macrophages incubated with both LPS and irradiated 4T1 cancer cells (10 Gy) (17) (**Figure 2**).

Enhanced radiation-induced NF-κB signaling can occur following activation of the apical DNA damage kinase, ATM. ATM-dependent NF-κB activation occurs following ubiquitination of NEMO (NF-κB essential modulator) which releases the cytoplasmic p50-p65 heterodimer allowing its translocation to the nucleus to act as a transcriptional activator (73).

ATM activation can also occur downstream of reactive oxygen species (ROS) production. NADPH oxidase 2 (NOX2)-dependent ROS production was reported to be important in ATM-dependent polarization of macrophages towards a pro-inflammatory phenotype through regulation of IRF5 at the mRNA and post-translational level. Therapeutically targeting other DNA damage response components, such as poly (ADP-ribose) polymerase (PARP) also appeared to activate macrophages towards a pro-inflammatory phenotype following increased ATM and IRF5 activation (74). Importantly enhanced expression of iNOS^+^CD68^+^ and NOX2^+^CD68^+^ TAMs was observed in resected specimens of rectal cancer patients with good responses to neoadjuvant radiotherapy (74). A recent study also suggested that targeting the angiogenic factor, fibroblast growth factor 2 (FGF2), in combination with radiotherapy can increase the iNOS^+^/CD206^+^ TAM ratio and improve tumor responses following fractionated radiotherapy (75). These data suggest that FGF2 could be considered as a therapeutic target to be exploited in combination with radiotherapy.

### Examples of potential barriers to effective polarization by radiation

As previously mentioned, radiotherapy induces the expression of CCL2 within the TME (28, 29). CCL2 acts to shift the recruited monocytes towards a more immunosuppressive phenotypic type directly by downregulating polarization-related gene expression and indirectly *via* T helper 2 cells (Th2) releasing anti-inflammatory cytokines such as IL-4, IL-6 and IL-10 (76). In a preclinical pancreatic ductal adenocarcinoma model, the inhibition of CCL2 in isolation had little impact on tumor growth unless used in combination with radiotherapy (77). It was found that irradiation of the tumor caused a significant increase in CCL2 production and radiation-dependent recruitment of monocytes/macrophages (77). Inhibiting this CCL2/CCR2 recruitment axis led to a decrease in tumor growth and vascularity (77). Additionally, the inhibition of CCL2 led to a decrease in TAM presence and a decrease in metastasis (78). This decrease in metastasis was caused by CCL2 inhibition reducing the production of CCL3 by immunosuppressive TAMs thereby reducing the ability of these macrophages to assist with tumor intravasation (78).

There has also been a lot of interest in therapeutically targeting CSF-1 signaling to modulate macrophage polarization following irradiation in a variety of cancers. In glioblastoma tumor models, CSF-1R inhibition delays recurrence following irradiation by reducing radiation-induced monocyte recruitment and differentiation to immunosuppressive TAMs (40). Interestingly, TAM survival in the context of CSF-1R inhibition appears to be facilitated by granulocyte-macrophage CSF (GM-CSF) and IFNγ (79). Altered TAM polarization and a reduction in macrophage migration was also seen in a preclinical prostate cancer model (38). Furthermore, in preclinical colorectal and pancreatic models, macrophage depletion using CSF-1 blocking antibodies, enhances the effectiveness of combined radiotherapy and immune checkpoint inhibitor (anti-PD-L1) treatment suggesting that macrophages act to hinder productive anti-tumor immune functions of radiotherapy (19).

Complement activation and signaling of complement anaphylatoxins through their respective receptors can also impact macrophage polarization. This is relevant in the context of radiotherapy since irradiation has been found to increase the local tumor expression of several complement factors in murine models (following 5 and 20 Gy irradiation) and in patient samples (treated with 1.5-2 Gy) (34). Of note, in the TME, the presence of stromal CD34^high^ fibroblasts expressing high levels of central complement component C3 (which when cleaved will result in C3a production) may also support the recruitment of macrophages with immunosuppressive phenotypes and results in attenuation of T-cell mediated responses (80). Interestingly, C3aR activation in TAMs can occur following intracellular production of C3a by tumor cells; and activation of PI3Kγ signaling downstream of C3aR activation contributes to suppression of anti-tumor responses (81). The effects of irradiation on intracellular C3a or C5a levels across tumor cells, however, is still unclear. Previously published work suggested that the presence of C5a and C3a might be essential for effective tumor radiation responses (34). However, the well-documented impact of C3a and C5a on macrophage recruitment and polarization towards immunosuppression may indicate that targeting the C3a-C3aR or C5a-C5aR signaling axes might prove to be beneficial in certain contexts. In combination with anti-PD-1 blocking antibodies, blocking C5a/C5aR1 signaling has indeed proven effective at improving primary and metastatic disease in lung tumor models (82). Similarly, in the B16-F10 melanoma model, blocking the PD-1/PDL-1 axis alongside C3a-C3aR or C5a-C5aR resulted in improved tumor control (83). The effects of radiotherapy in combination with immune checkpoint and C5a/C5aR1 inhibition, however, has yet to be determined.

The use of TGF-β inhibition in combination with PD-1/PD-L1 inhibition has also found success in a multitude of clinical trials, with phase two trials commencing in non-small cell lung (NCT03631706), triple negative breast (NCT03579472), colorectal (NCT03724851), and pancreatic (NCT02734160) cancers. A summary of additional recent clinical trials combining radiotherapy and macrophage targeting is shown in **Table 1**. Interestingly, combining TGF-β and PD-1 inhibition with radiotherapy in a preclinical colorectal cancer model demonstrated improved survival plus reduced tumor growth (84). Additionally, this study demonstrated a reduction in TAM recruitment to both primary tumors as well as non-irradiated bilateral lesions (84).

**Table 1:** Table summarizing latest clinical trials combining radiotherapy and approaches which may impact macrophage recruitment or function.

Target	Drug	Combination	Cancer Type	Phase	Year	Reference
ATM	AZD1390	RT	Glioblastoma	I	2018	NCT03423628
CD47/SIRPα	RRx-001	RT + Temozolomide	Gliomas	I	2016	NCT02871843
CD40	CDX-1140	RT + Poly-ICLC + FLT3-L	Breast	I	2020	NCT04616248
CSF-1R	Cabiralizumab	RT + Nivolumab	Pan-	I	2018	NCT03431948
	Sunitinib	RT	Head and Neck, Pelvic, Nervous System, Thoracic	I	2007	NCT00437372
	Sunitinib	RT	Metastatic	I/II	2007	NCT00463060
	Sunitinib	RT	Soft Tissue Sarcoma	I/II	2008	NCT00753727
	Sunitinib	RT	Glioblastoma	II	2010	NCT01100177
	Sunitinib	RT + Temozolomide	Glioblastoma Multiforme	II	2016	NCT02928575
	Sunitinib	RT + Surgery + Irinotecan + Cisplatin	Esophageal	II	2006	NCT00400114
	Sunitinib	RT + Leuprolide + Goserelin + Casodex	Prostate	I	2008	NCT00631527
	Nilotinib	RT	Chordoma	I	2011	NCT01407198
	PLX3397	RT + Temozolomide	Glioblastoma	I/II	2013	NCT01790503
	PLX3397	RT + Anti-hormone Therapy	Prostate	I	2015	NCT02472275
CCR2/CCR5	BMS-813160	RT + Nivolumab + GVAX	Pancreatic Ductal Adenocarcinoma	I/II	2018	NCT03767582
PI3Kγ	BYL719	RT + Cetuximab	Head and Neck Squamous Cell	I	2014	NCT02282371
	BYL719	RT + Cisplatin	Head and Neck Squamous Cell Carcinoma	I	2015	NCT02537223
	BKM120	RT + Temozolomide	Glioblastoma	I	2011	NCT01473901
	BKM120	RT + Cisplatin	Multiple	I	2014	NCT02113878
TLR3	Poly-ICLC	RT + Temozolomide	Glioblastoma Multiforme	II	2005	NCT00262730
TLR7/9	Imiquimod	RT + Cyclophosphamide	Breast	I/II	2011	NCT01421017
TLR9	SD-101	RT	B-Cell Lymphoma	I/II	2014	NCT02266147
	SD-101	RT + Ibrutinib	Follicular Lymphoma	I/II	2016	NCT02927964
	SD-101	RT + Nivolumab	Pancreatic	I	2019	NCT04050085

Search conducted on ClinicalTrials.gov using search criteria “Cancer”, “Radiation” and “Macrophage”. CSF-1R, Colony Stimulating Factor Receptor 1; CCR2, C-C Chemokine Receptor; PI3Kγ, Phosphatidylinositol-4,5-bisphosphate 3-kinase catalytic subunit gamma; TLR, Toll-Like Receptor; RT, Radiotherapy.

## Conclusion

Effectively modulating the immunostimulatory effects of radiation has the enticing potential to improve local and distant tumor control (85). Given the relatively high numbers of macrophages in the TME (relative to other cell types) and the enhanced macrophage recruitment observed following irradiation, it is likely that combination therapies will have to consider how to polarize this immune population to the pro-inflammatory, tumoricidal side of the spectrum (86). Indeed, investigation into targeting TAMs is currently at the forefront of cancer immunotherapies and, a greater understanding of mechanisms of recruitment and pro-tumor activity of these macrophages may provide new therapeutic opportunities to improve the efficacy of existing treatments (39). Targeting the soluble factor-receptor axes interactions that may pose a barrier to the most effective polarization could be considered. For example, CSF1-CSF1R, C5a-C5aR1, FGF2 or TGFβ/TGFβR blockade in combination with immune checkpoint inhibitors such as PD1/PDL-1 could be promising strategies (19, 84). Further research into the effect of different radiation doses and fractionation regimes on macrophage recruitment and plasticity will help optimize the timing and nature of the most effective combination therapies. A consideration of the effect of an altered macrophage response to normal tissue toxicity following radiotherapy will also be important since maximal therapeutic benefit relies on effective tumor control with minimal normal tissue toxicity.

## Author contributions

Conceptualization: CB and MO. Writing original draft: CB, DMac, DMaj, and MO. Writing review and editing: CB, DMac, DMaj, JA, and MO. Resources: JA and MO. Supervision: JA and MO. Funding acquisition: JA and MO. All authors contributed to the article and approved the submitted version.

## Funding

This work was supported by the Medical Research Council (MC_UU_00001/10). C.B is supported by an International Accelerator Award, ACRCelerate funded by Cancer Research UK (A26825 and A28223). J.N.A. is the recipient of a Cancer Research Institute/Wade F.B. Thompson CLIP grant (CRI3645) and Cancer Research UK grant DCRPGF\100009.

## Acknowledgments

We apologies to all the authors that we could not cite due to space constraints. This work was supported by the Medical Research Council (MC_UU_00001/10). C.B is supported by an International Accelerator Award, ACRCelerate funded by Cancer Research UK (A26825 and A28223). J.N.A. is the recipient of a Cancer Research Institute/Wade F.B. Thompson CLIP grant (CRI3645) and Cancer Research UK grant DCRPGF\100009.

## Conflict of interest

The authors declare that the research was conducted in the absence of any commercial or financial relationships that could be construed as a potential conflict of interest.

## Publisher’s note

All claims expressed in this article are solely those of the authors and do not necessarily represent those of their affiliated organizations, or those of the publisher, the editors and the reviewers. Any product that may be evaluated in this article, or claim that may be made by its manufacturer, is not guaranteed or endorsed by the publisher.
